# Psychedelic Communitas: Intersubjective Experience During Psychedelic Group Sessions Predicts Enduring Changes in Psychological Wellbeing and Social Connectedness

**DOI:** 10.3389/fphar.2021.623985

**Published:** 2021-03-25

**Authors:** H. Kettner, F. E. Rosas, C. Timmermann, L. Kärtner, R. L. Carhart-Harris, L. Roseman

**Affiliations:** ^1^ Centre for Psychedelic Research, Department of Brain Sciences, Imperial College London, London, United Kingdom; ^2^ Data Science Institute, Imperial College London, London, United Kingdom; ^3^ Centre for Complexity Science, Imperial College London, London, United Kingdom

**Keywords:** psychedelics, social connectedness, wellbeing, communitas, collective experience, mental health, set and setting, ceremony

## Abstract

**Background:** Recent years have seen a resurgence of research on the potential of psychedelic substances to treat addictive and mood disorders. Historically and contemporarily, psychedelic studies have emphasized the importance of contextual elements ('set and setting') in modulating acute drug effects, and ultimately, influencing long-term outcomes. Nevertheless, current small-scale clinical and laboratory studies have tended to bypass a ubiquitous contextual feature of naturalistic psychedelic use: its social dimension. This study introduces and psychometrically validates an adapted *Communitas Scale,* assessing acute relational experiences of perceived togetherness and shared humanity, in order to investigate psychosocial mechanisms pertinent to psychedelic ceremonies and retreats.

**Methods:** In this observational, web-based survey study, participants (*N* = 886) were measured across five successive time-points: 2 weeks before, hours before, and the day after a psychedelic ceremony; as well as the day after, and 4 weeks after leaving the ceremony location. Demographics, psychological traits and state variables were assessed pre-ceremony, in addition to changes in psychological wellbeing and social connectedness from before to after the retreat, as primary outcomes. Using correlational and multiple regression (path) analyses, predictive relationships between psychosocial 'set and setting' variables, communitas, and long-term outcomes were explored.

**Results:** The adapted Communitas Scale demonstrated substantial internal consistency (Cronbach's alpha = 0.92) and construct validity in comparison with validated measures of intra-subjective (visual, mystical, challenging experiences questionnaires) and inter-subjective (perceived emotional synchrony, identity fusion) experiences. Furthermore, communitas during ceremony was significantly correlated with increases in psychological wellbeing (*r* = 0.22), social connectedness (*r* = 0.25), and other salient mental health outcomes. Path analyses revealed that the effect of ceremony-communitas on long-term outcomes was fully mediated by communitas experienced in reference to the retreat overall, and that the extent of personal sharing or ‘self-disclosure’ contributed to this process. A positive relationship between participants and facilitators, and the perceived impact of emotional support, facilitated the emergence of communitas.

**Conclusion:** Highlighting the importance of intersubjective experience, rapport, and emotional support for long-term outcomes of psychedelic use, this first quantitative examination of psychosocial factors in guided psychedelic settings is a significant step toward evidence-based benefit-maximization guidelines for collective psychedelic use.

## Introduction

The ‘biomedical revolution' of the 1970 and 80s led to an explosive infiltration of pharmaceutical interventions into psychiatric practice and the marginalization of previously favored psychodynamic approaches, consequentially inviting the charge that clinical psychopharmacology offers an atomized and decontextualized approach to mental illness, improving revenues rather than mental health outcomes or stigma (Deacon, 2013; Kleinman, 1988; Kleinman and Cohen, 1991; Peele, 1981). Research on the therapeutic application of psychedelic serotonin 2A receptor agonists (such as LSD or psilocybin) constitutes a curious outlier in this regard, considering the strong emphasis it places on the role of psychological, social, and cultural context of administration–popularly known as ‘set and setting'–for attaining therapeutic success (Leary et al., 1963; Hartogsohn, 2016; Hartogsohn, 2017; Carhart-Harris, 2018; Carhart-Harris et al., 2018b). However, motivated by pragmatic and safety reasons, modern trials of psychedelic-assisted psychotherapy have almost exclusively employed individual, rather than systemic or group therapy approaches–consistent with dominant psychedelic therapy models of the 1950s–70s (Grof, 1980), although modern (Anderson et al., 2019) and historical group-therapy exceptions do exist (see Trope et al., 2019 for a historical review of psychedelic-assisted group therapy research). This focus on individual therapy, delivered in highly engineered clinical and laboratory settings, might partially explain why contemporary psychedelic research has remained largely silent in relation to what might be one of the most prevalent factors impacting psychedelic effects *in natura*: the social dimension of psychedelic use.

The socially constructive function of psychedelic use has been central in many cultures that developed customary or ritualized forms of consumption (Dobkin de Rios, 1984; Andritzky, 1989; Hunt and Barker, 2001; Winkelman, 2010), and can be deduced even from earliest mentions and anthropological studies of indigenous usage (Villavicencio, 1858; La Barre, 1938). An inherently social function is also reflected in the plethora of approaches to psychedelic use that persists in contemporary Western cultures, ranging from dance-events (“raves”) (Papadimitropoulos, 2009) to religious ceremonies conducted by ayahuasca churches (Tupper, 2008), to the medically supervised administration of ibogaine in underground addiction treatment centres (Alper et al., 2008). The number of people drawing on various types of these naturalistic settings by consuming psychedelic substances on their own behalf is increasing (Palamar and Le, 2018; Yockey et al., 2020), often with self-medicative or self-explorative purposes (Mason and Kuypers, 2018; Kettner et al., 2019b; Hutten et al., 2019). Importantly, naturalistic settings in which psychedelics are taken almost always involve their use as a collective activity, raising the question of how psychedelic substances may acutely affect the experience of intersubjectivity, i.e., human interaction, relation, and collective emotion, and how such psychosocial dynamics might, in turn, act upon the psychological constitution of the individual.

A rapidly growing phenomenon that lends itself particularly well to the study of psychosocial effects of psychedelics can be found in psychedelic retreat settings. In countries where specific psychedelic substances have remained legal, the unmet global demand for structured and safe use of psychedelics has helped create an industry of psychedelic experience-provision, often comprising of multi-day retreat programs, typically consisting of one or more guided psychedelic group sessions, referred to as ‘ceremonies’. Usually, such guided psychedelic sessions are prepared and conducted by one or more individuals who have acquired this skill through formal training or experience in the care for others during psychedelic experiences. These practitioners (e.g. ‘shamans’, ‘ayahuasceros’ or ‘curanderos’ in the context of ayahuasca ceremonies; ‘experience facilitators’, ‘sitters’ or ‘guides’ in Western settings) may carry out or recommend preparatory activities before the session to induce a prepared mind-“set” in the participants (Harris and Gurel, 2012). Additionally, they strive to establish a safe and comfortable environment, often making use of decorative or ritual objects, special lighting, incense or bonfires, and provide emotional support to participants under the influence of the psychedelic (Fadiman, 2011). During the session, facilitators or shamans may interact with participants directly or indirectly, but most often including the medium of music. Rattling, drumming, singing and whistling, sometimes including the group’s active participation, are typical elements in Meso and South American settings of mescaline (i.e. peyote or San Pedro) and ayahuasca use (De Rios and Katz, 1975).

While many psychedelic retreat providers in the West utilize elements discovered to be useful in psychedelic therapy such as tailored playlists for psychedelic sessions (Kaelen et al., 2018), most facilitators also draw from other therapeutic or spiritual traditions. These often include techniques aimed to structure the relational dynamics amongst retreat participants and between participants and facilitators. A prevalent example are ‘sharing-round’ rituals (Fotiou, 2020; Gearin, 2015a; Gearin, 2016b), often held both before and after ceremonies, during which participants are invited to articulate personally salient material, such as intentions for, or experiences during sessions. Controlled self-disclosure of feelings and thoughts, being fundamental for developing social relationships (Derlega and Chaikin, 1977), has been shown to elicit trust and reciprocity especially among 'passing strangers' (coming from different locations) (Rubin, 1975) and liking within groups (Collins and Miller, 1994). These ritualized and carefully moderated spaces for exchange may therefore facilitate the creation of intragroup cohesion and alliance with facilitators, the potential importance of which, although not yet investigated empirically, can best be understood in light of group psychotherapy research, where cohesion and therapeutic alliance are among the most powerful predictors of positive treatment-outcomes (Schnur and Montgomery, 2010). On an individual level, apart from rendering facilitators potentially more able to adequately react to difficult material arising during psychedelic sessions, disclosure of deeply personal, especially challenging, narratives or experiences, without others advising or judging, has been shown to improve emotion regulation and enhance self-acceptance (Farber, 2003; Hemenover, 2003; Kahn and Garrison, 2009), suggesting that sharing-rounds in psychedelic retreats might in themselves hold therapeutic value (Aronovich, 2020).

In addition to the relational processes commonly employed in preparation and aftercare of ceremonial psychedelic use, one can also expect the acute psychedelic state itself to be impacted by the presence of others. However, little to no quantitative empirical research exists on the intersubjective phenomenology of psychedelic states, which is striking considering the substantial body of literature indicating a crucial role of the acute psychedelic state mediating the long-term psychological outcomes of psychedelic substance use (MacLean et al., 2011; Uthaug et al., 2018a; Haijen et al., 2018; Roseman et al., 2018; Kettner et al., 2019a; Roseman et al., 2019; Uthaug et al., 2019; Spriggs et al., 2020). Whereas psychedelic research has so far focused largely on individual, i.e., intrasubjective drug effects, entry points into relational or ‘intersubjective’ aspects of shared altered states of consciousness can be found in studies on ritual and collective action. As proposed by Durkheim (1897), an important function of collective dancing, music listening, or music-making (which are commonly also employed in psychedelic group settings) lies in their ability to induce a synchronization of affect and behavior among participants. This phenomenon, originally termed 'collective effervescence' (Durkheim, 1912) has been identified as a catalyst for the blurring of self-other boundaries, social affiliation and cooperation (Hove and Risen, 2009; Reddish et al., 2013; Tarr et al., 2014). Specifically, Páez et al. (2015) found increased perception of emotional synchrony in collective gatherings to elicit ‘identity fusion’, a process that implies a change in self-concept from a personal to a collective level. Identity fusion has been described as “a unique form of alignment with a group, one that entails a visceral feeling of oneness with the group” (Swann et al., 2012), which, in concurrence with phenomena of synchrony, may explain how collective rituals often reinforce social connectedness and cohesion (Whitehouse et al., 2014).

The theory of identity fusion is conceptually linked to Victor Turner, (1969) notion of “spontaneous communitas”, defined as an experience of intense togetherness and shared humanity that temporarily transcends social structures. According to Turner, communitas occurs in situations such as rites of passage, where existing relational structures are lifted in order to allow for a transition into a new social configuration, mediated by an anti-structural and often ritualized 'liminal phase' of equality among community members. Communitas, thereby, involves a transgression or dissolution of norms that regularly govern structured or institutionalized relationships, placing the group that experiences it temporarily 'outside' of society. Unsurprisingly, modern conceptions of communitas thus also include states of spontaneous fellowship during events such as natural disasters, revolutions, countercultural happenings, music or sport events (Turner, 2012), which suspend conventional social structures. Explaining how communitas may generate enduring changes beyond the acute liminal state, Olaveson (2001) discusses how the dialectic of communitas lies in the revitalizing function that this anti-structural state entails, owed to its creative potential: "In liminality, the state in which communitas occurs, culture is analyzed into factors and freely recombined and experimented with (Turner and Turner, 1970) […] Anti-structure can thus be a positive and generative force (Turner, 2018), and is a condition in which myths, symbols, rituals, philosophy, and art are generated, which are templates for the periodical reclassification of reality and man's relationship to society, nature, and culture." (Olaveson, 2001, *p*. 106).

The concept of communitas thereby invokes a fascinating semblance to the transformative potential of psychedelic states, which analogously has been discussed as resulting from a temporary suspension of established hierarchical neurocognitive structures, allowing for less constrained recombination of thoughts, emergence of novel insights, and sometimes radical reorientation of the individuals' relationship to themselves, others, and reality at large (Carhart-Harris and Friston, 2019). While seeing the psychedelic state as a 'functional communitas of the brain' might be an overly stretched analogy, Turner's concept of spontaneous communitas has indeed been applied to explain transformative collective experiences both in psychedelic rave culture (Tramacchi, 2000), as well as in psychedelic ceremonies (Lewis, 2008; Mantere, 2013; Roseman et al., 2021), although not yet assessed quantitatively in this context. Communitas as a sociopsychological construct has so far only been operationalized in the contexts of sports (McGinnis et al., 2012) and night clubbing (Taheri et al., 2016), finding that communitas as an intersubjective experience was more relevant than the intrasubjective experience of 'flow' in the context of night clubbing, but not golfing, for the generation of emotional responses, and enduring involvement, respectively. Of note, both studies showed discriminant validity of their respective measure of Communitas only against intra- but not related intersubjective (e.g., identity fusion, emotional synchrony) constructs.

The current study aimed to address the existing gap of knowledge and research instruments pertaining to relational processes pertinent to psychedelic experiences, providing a first comprehensive, quantitative assessment of psychosocial mechanisms underlying collective psychedelic use. For this purpose, we here leverage the largest prospective sample of psychedelic use reported to date to psychometrically validate an adapted psychedelic *Communitas Scale* (COMS), a measure of communitas specifically tailored to psychedelic group experiences. Construct and criterion validity are investigated through the COMS′ relationship to: 1) validated measures of intersubjective experience and acute psychedelic effects; 2) 'set and setting', specifically its relational elements (identity fusion, self-disclosure, rapport with participants and facilitators) and trait absorption, a well-established predictor of previously studied intrasubjective psychedelic effects (Studerus et al., 2012; Haijen et al., 2018); as well as 3) long-term psychological outcomes, including psychological wellbeing and social connectedness as primary outcomes, and depressive symptoms, trait anxiety, and interpersonal tolerance as secondary outcomes. This was achieved by means of correlational and longitudinal path analyses, the latter approach being able to reveal directed causal dependencies between variables measured at multiple timepoints.

## Methods

The current study used web-based data collection from a self-selected opportunistic volunteer sample. Eligibility criteria for participants were being 18 years or older, a good comprehension of the English language, and the intention to participate in a retreat, ceremony, or other guided experience involving the use of a classic psychedelic containing a 5-HT2A receptor agonist (e.g., psilocybin, DMT, mescaline, or LSD). Ethics approval was granted by the Joint Research Compliance Office and the Imperial College Research Ethics Committee (ICREC reference 18IC4346). The online survey platform *Surveygizmo* was used to create and host the survey.

Participants were recruited following two dissemination strategies: Firstly, advertisements including a link to the study website (www.ceremonystudy.com) were posted and shared on psychedelic-related online for a (Reddit groups, Bluelight, and ayahuasca. nl), email newsletters (MAPS, Chakruna, and psychedelicexperience.net), and social media platforms (Twitter and Facebook). Secondly, retreat centers and facilitators of guided psychedelic experiences were contacted with information about the study, inviting them to advertise the study to their future clients. This mediated outreach strategy was chosen to maximize awareness of the study in the target population, and to facilitate the collective participation of multiple participants from single retreat or ceremony groups.

### Study Design and Timepoints

In this prospectively designed study, a minimum of five surveys were completed by participants at different time points. A first baseline measurement took place 2 weeks before the psychedelic experience, followed by a second measurement up to 3 h before. A third measurement took place on the day after the experience, in which subjective drug effects were assessed retrospectively.^1^ A fourth survey was completed on the day after leaving the ceremony or retreat location. Finally, the fifth, key endpoint survey was completed 4 weeks after the end of the psychedelic retreat or ceremony.

Each survey included a number of existing measures and in some cases self-constructed scales to assess particular constructs of interest for which no previously validated measures could be found. The following section describes the measures that were included in the present analysis.

### Measures

#### Baseline Measurement (Survey 1)

##### Participant Information

The baseline survey included questions assessing demographic information, including age, gender, nationality, education, employment, income, ethnicity, and marital status, as well as frequency of lifetime psychedelic use.

##### Experience Details

Next, a number of questions served to specify details of the upcoming experience. These included the type of plant or substance expected to be used (“Psilocybin/magic mushrooms/truffles”; “LSD/1P-LSD”; “Ayahuasca”; “DMT”; “5-MeO-DMT (*Bufo*)”; “San Pedro”; “Peyote”; “Iboga/Ibogaine”; self-specified option), the duration (in days) and number of psychedelic sessions of the retreat.

##### Outcome Measures

As primary outcomes, the 14-item Warwick–Edinburgh Mental Wellbeing Scale (WEMWBS) (Tennant et al., 2007) and the 8-item Social Connectedness Scale (SCS) (Lee and Robbins, 1995) were used to assess longitudinal changes in wellbeing and psychosocial health from before to after the psychedelic experience. Of secondary interest, the self-report version of the quick inventory of depressive symptomatology (QIDS-SR-16, hereon referred to as the QIDS) (Rush et al., 2003) and the 6-item short form of the Spielberger Trait-State Anxiety Inventory (STAI) (Fioravanti-Bastos et al., 2011) trait subscale were included to measure changes in depressive symptoms and trait anxiety. Lastly, the Warm Tolerance subscale of the Interpersonal Tolerance Scale (IPTS) (Thomae et al., 2016) was included to measure changes in attitudes and openness toward people who hold different values or beliefs than one's own.

##### Psychological Traits

An established trait predictor of psychedelic effects (Haijen et al., 2018; Lifshitz et al., 2019), trait absorption was measured using 25 items of the Modified Tellegen Absorption Scale (MODTAS) (Jamieson, 2005), a previously identified predictor of acute psychedelic drug effects (Studerus et al., 2012; Haijen et al., 2018).

#### Pre-experience Measurement (Survey 2)

##### Preparedness—“Set”

The 12-item psychedelic predictor scale (Haijen et al., 2018) was used to assess preparedness for the experience. A sub-score for the social setting pre-ceremony (from hereon referred to as 'Rapport') was calculated as a sum of the items "*I have a good feeling about my relationship with the group/people who will be with me during the experience*" and "*I have a good relationship with the main person/people who will look after me during the upcoming experience*" from the psychedelic predictor scale. In the present sample, the Pearson correlation coefficient for the two items of this subscale was strong (*r* = 0.62). Additionally, a single-item pictographic identity fusion scale (IF) (Swann et al., 2009) was included to assess identity fusion with the group shortly before the session.

#### Post-experience Measurement (Survey 3)

##### Subjective Psychedelic Experience

To assess subjective drug effects, a combination of validated and self-constructed questionnaires was employed. These included: In order to measure visual perceptual alterations, the three subscales on visual effects of the Altered States of Consciousness Questionnaire (ASC-VE) were included: elementary imagery, complex imagery and audio-visual synaesthesia (Studerus et al., 2010); the 30-item Mystical Experience Questionnaire (MEQ) (Barrett et al., 2015), including four subscales (mystical, positive mood, transcendence of time and space, ineffability); the 25-item Challenging Experience Questionnaire (CEQ) (Barrett et al., 2016) comprising seven subscales (fear, grief, physical distress, insanity, isolation, death, paranoia); and an adapted short 5-item version of the Perceived Emotional Synchrony Scale (PESC) (Páez et al., 2015), assessing feelings of collective emotional entrainment. To further assess the psychosocial dimensions of the experience, the pictographic IF was included retrospectively to assess perceived fusion with the group during the experience (see Appendix). Lastly, an 8-item Communitas Scale (COMS, see Appendix) was adapted based on items previously used in the context of sports (McGinnis et al., 2012) and night clubs (Taheri et al., 2016), rated on a 7-point Likert scale. Specifically, the 5 items used by Taheri et al. (2016) were reworded to apply to psychedelic ceremonies, rather than night clubs. Also, three additional items were constructed to capture aspects of Communitas that were deemed central to the construct based on the primary literature, but not captured in above-mentioned previous operationalisations, i.e., irrelevance of social status, experience of equality, loss of ego.

Two further self-constructed items were added, which assess the connection felt with another participant and with a facilitator during the experience, respectively. In order to assess the impact of emotional support delivered in the ceremonial setting, participants rated one further self-constructed item “*To what extent did the presence of emotionally supportive individuals influence the overall quality of your experience?*" on a 1–100 VAS.

#### Post-retreat (Survey 4)

##### Retreat Experience

On the day after the retreat, an adopted version of the COMS and pictographic IF scale were included, measuring both constructs in reference to the retreat as a whole, rather than just the duration of a single psychedelic ceremony. Additionally, the revised self-disclosure scale (RSDS) (Wheeless and Grotz, 1976) was used to measure self-perceived self-disclosure across six components (intended disclosure, positiveness, honesty, amount, relevance, and depth of disclosure), using a total of 18 items rated on a 7-point Likert scale which are summed into a total self-disclosure score.

#### Key Endpoint (Survey 5)

##### Outcome Measures

In order to assess changes on primary and secondary outcomes, measures assessing psychological wellbeing (WEMWBS), social connectedness (SCS), depressive symptoms (QIDS), trait anxiety (STAI), and interpersonal tolerance (IPTS) were repeated at the key endpoint, 4 weeks post-experience.

### Statistical Analysis

Data from all time points was imported and merged using the Statistics Toolbox or Matlab (release 2019b) and were then exported for further analysis into RStudio (v1.2). Several heuristics were employed to first verify the factor structure and internal consistency among the eight items of the COMS. Since, in accordance with its previous versions (McGinnis et al., 2012; Taheri et al., 2016), we hypothesized the COMS to be unidimensional, a confirmatory factor analysis was carried out after verifying the number of latent factors, as recommended by Matsunaga (2010). Visual examination of the scree plot and the Kaiser, (1960), which accepts as reliable factors those whose corresponding eigenvalue is larger than one, were applied. Complementary analyses included, optimal coordinate and acceleration factor tests in order to identify an appropriate number of factors via non-graphical solutions (Raîche et al., 2013). The scale reliability was assessed via Cronbach’s alpha and composite reliability—the latter being less prone to over- or underestimations of reliability at a population level (Raykov, 1998).

#### Construct Validity

To assess construct validity, bivariate Pearson correlations were calculated between COMS scores, self-constructed items on participant and facilitator-connectedness, and the subscales of validated subjective experience measures taken at each ceremony (IF, PESC, MEQ, CEQ, ASC-VE).

#### Criterion Validity

To test the primary hypothesis—that communitas experienced during ceremony would be predictive of changes in the primary outcomes wellbeing (WEMWBS) and social connectedness (SCS)—Pearson-correlations were calculated between SCS, WEMWBS and COMS scores. For participants who reported multiple ceremonies, the analyses considered the highest COMS score reported. Additionally, two-sided pairwise t-tests were conducted for SCS and WEMWBS to assess significance of changes from baseline to the key endpoint at 4 weeks post-retreat. The same procedure was repeated for the secondary outcomes trait anxiety (STAI), depression severity (QIDS), and interpersonal tolerance (IPTS).

#### Path Analysis

**FIGURE 1:**
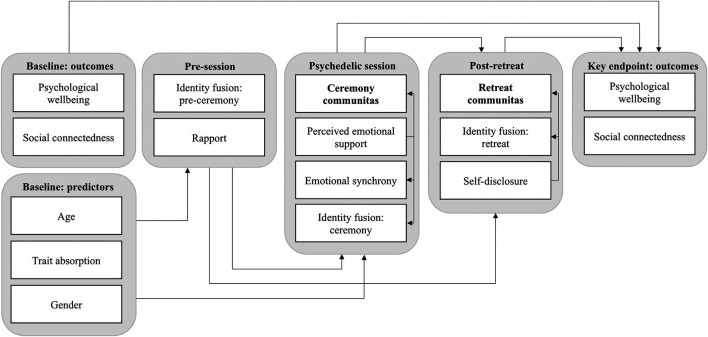
Initial path model showing hypothesized relationships of psychosocial variables during settings of collective psychedelic use, measured across 5 time points.

In order to further explore the causal structure of the assessed psychosocial factors and examine the concurrent, pre- and postdictive criterion validity of the COMS, a longitudinal path analysis was conducted across the five collected timepoints, including individuals who partook in no more than one ceremony over the course of a retreat (N in analysis = 631). As a first step, an initial model was constructed considering the following variables: 1) Psychological trait absorption and demographics (age, gender) measured at baseline; 2) Identity fusion and rapport assessed hours before the psychedelic session; 3) Perceived emotional synchrony, communitas, identity fusion and 'perceived emotional support' during the session, assessed on the following day; 4) Self-disclosure, communitas and identity fusion in relation to the retreat as a whole, measured on the day after leaving the ceremony location; and 5) Psychological well-being and social connectedness as long-term psychological outcome variables, assessed 4 weeks later and controlled for baseline variables. In this initial model (represented in Figure 1), regression paths were set so that each variable at time point *t* would predict each variable at time points *t* + 1 and *t* + 2, thereby accounting for uncaptured delayed effects. Additionally, theoretically motivated concurrent regression paths were allowed within timepoints 3) and 4) from 'perceived emotional support' and 'self-disclosure' to the remaining variables within the respective timepoint.

As a second step, an iterative model pruning process was employed that removed the following elements: 1) Non-significant paths, and 2) Variables that exhibited no significant direct or indirect effects on either communitas or any of the outcome measures, psychological wellbeing (WEMWBS) and social connectedness (SCS), were dropped from the model, until all remaining effects were significant at *p* < 0.05. Estimation methods were adjusted during the pruning process, using weighted least squares (WLS) estimation where endogenous non-continuous variables were present, and robust maximum likelihood estimation (MLR) when all endogenous variables were continuous. As recommended by Kline (2015), several indicators of overall fit are reported for the initial and final model, including the model Chi-Square, Root Mean Square Error of Approximation (RMSEA), Comparative Fit Index (CFI), and Standardized Root Mean Square Residual (SRMR). Cut-off values for determining fit quality (presented in Table 1) were based on previous literature (Hooper et al., 2008; Hair et al., 2010; Awang, 2012).

**TABLE 1:** Recommended and actual fit indices for initial and pruned path models.

Fit index	Initial model	Final model	Good fit	Acceptable fit
CFI	0.926	0.974	>0.95	>0.90
χ^2^/df	2.70	1.45	<2.0	<3.0
RMSEA	0.090	0.049	<0.05	<0.08
SRMR	0.136	0.068	<0.05	<0.08
Estimator	WLS	MLR		

CFI: Comparative Fit Index, χ2: Chi-square test statistic, df: degrees of freedom, RMSEA: Root Mean Square Error of Approximation, SRMR: Standardized Root Mean Square Residual, WLS: Weighted Least Squares, MLR: Robust Maximum-Likelihood.

Following Acock (2014), effects strengths were interpreted based on standardized beta coefficients, where *β* < 0.2 is considered a weak, 0.2< *β* < 0.5 moderate, and *β* > 0.5 a strong effect.

## Results

### Demographic information

At the time of analysis, data was collected from a total of N = 886 participants. In comparison, the surveys were completed by N_1 (Baseline)_ = 819; N_2 (Pre-session)_ = 582; N_3 (Post-session)_ = 533; N_4 (Post-retreat)_ = 432; N_5 (4-week endpoint)_ = 399 participants. Of note, time point N_3 (Post-session)_ was additionally completed for a second and a third session by 97 and 89 participants, respectively, resulting in a total of 720 observations of acute psychedelic effects experienced during a group session. Demographic information collected during the baseline survey are presented in Table 2. Demographic information of participants remaining at time point 3 (post-ceremony), is provided in Supplementary Table S1.

**TABLE 2:** Demographic information collected at baseline.

Total *N*	819
Age	44.4 ± 12.6
Gender	
Female	359 (43.8%)
Male	455 (55.6%)
Other	5 (0.6%)
Nationality	
United States	359 (43.8%)
United Kingdom	160 (19.5%)
Australia	31 (3.8%)
Germany	28 (3.4%)
Canada	26 (3.2%)
Other countries (53 in total)	215 (26.3%)
Education	
None	6 (0.7%)
High School or equivalent (GED)	62 (7.6%)
Associate/Technical Degree	58 (7.1%)
College diploma	250 (30.1%)
Master’s degree	275 (33.6%)
Doctorate or professional degree	168 (20.5%)
Employment	
Student	46 (5.6%)
Working full-time	520 (63.4%)
Working part-time	120 (14.7%)
Retired	73 (8.9%)
Unemployed	60 (7.3%)
Median household income	9,000 $/month
Ethnicity	
White	743 (90.7%)
Black or African American	12 (1.5%)
Asian	48 (5.9%)
American Indian or Alaska native	3 (0.4%)
Unknown/Prefer not to say	11 (1.3%)/23 (2.8%)
Marital status	
Cohabiting with partner	101 (12.3%)
Married	340 (41.5%)
Divorced	86 (10.5%)
Separated	29 (3.5%)
Never married	254 (31.0%)
Widowed	9 (1.1%)
Previous psychedelic use	
Never	330 (40.3%)
Once	95 (11.2%)
2–5 times	166 (20.3%)
6–10 times	73 (8.9%)
11–20 times	76 (9.3%)
21–50 times	49 (6.0%)
>50 times	30 (3.7%)

Details about the retreat duration, number of planned ceremonies, and substance used are shown in Table 3. Psilocybin mushrooms/truffles (80.0%) and ayahuasca (15.9%) jointly made up for 95.9% of the substances that participants were planning to use during their experience.

**TABLE 3:** Retreat details collected at baseline.

Substance used	Psilocybin/Magic mushrooms/truffles	656 (80.0%)
	Ayahuasca/Yagé	130 (15.9%)
	Other substance	33 (4%)
Retreat duration	1 day	279 (34.0%)
	2 days	20 (2.4%)
	3 days	154 (18.8%)
	4–6 days	200 (24.4%)
	7 days	180 (22.0%)
	8 or more days	46 (5.6%)
Psychedelic sessions	1 session	466 (56.9%)
	2 sessions	109 (13.3%)
	3 sessions	199 (24.2%)
	4 or more sessions	45 (5.5%)

Absolute frequencies including corresponding percentages (in brackets) are presented.

### Communitas Scale Psychometric Properties

#### COMS Internal consistency

**FIGURE 2:**
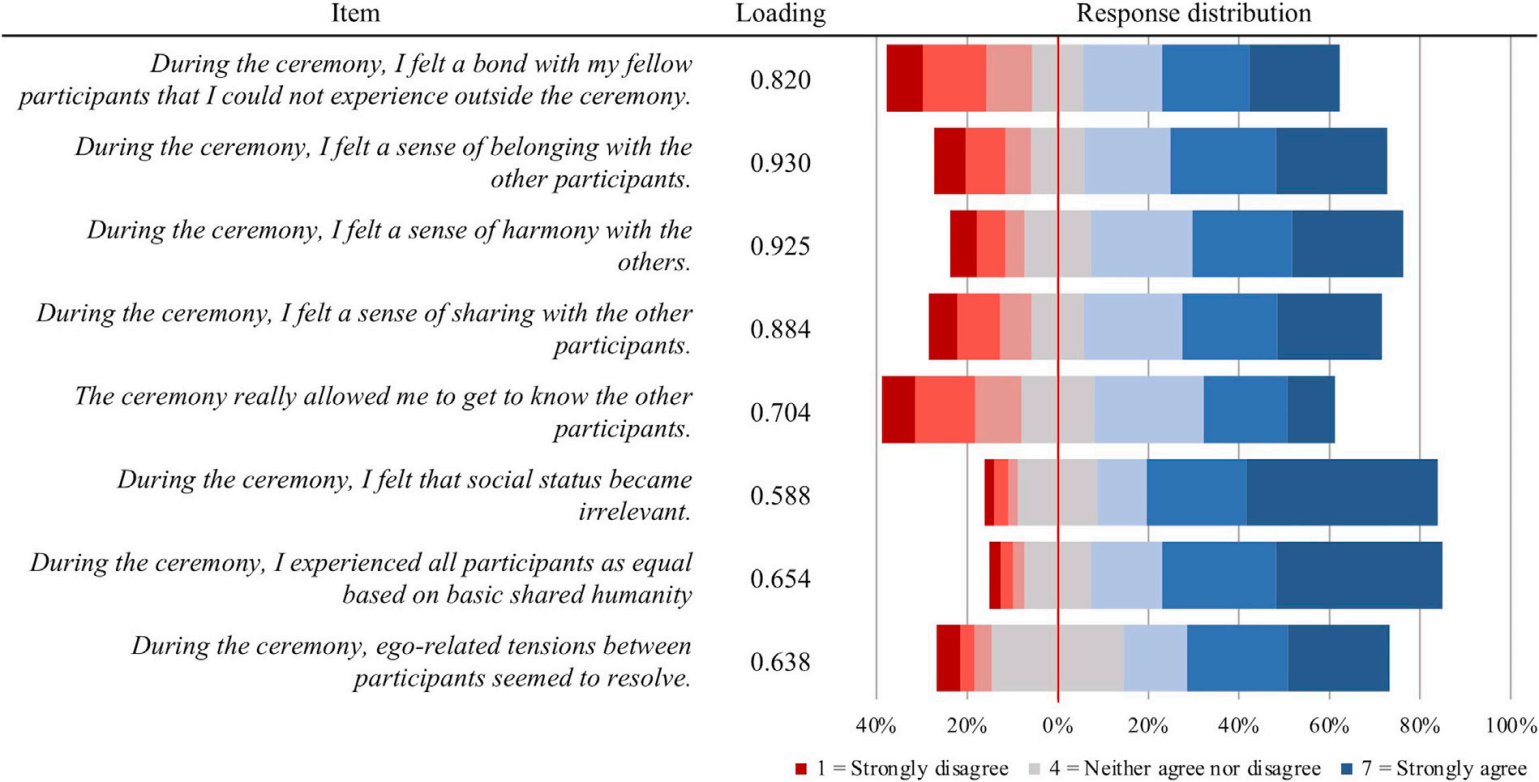
Items, factor loadings, and response distributions of the adopted Communitas Scale (COMS). Loadings refer to standardized factor loadings based on a single-factor confirmatory factor analysis.

All the considered approaches to determine the optimal number of factors to retain from the COMS pointed toward a unifactorial solution across the 8 items (Supplementary Figure S1), with an eigenvalue of 5.23 for the first factor which explained 65.7% of total variance in the data. Internal consistency of this single factor solution was excellent with Cronbach’s alpha = 0.92. As shown in Figure 2, single-factor confirmatory factor analysis revealed that the standardized factor loadings for all items were above the recommended threshold of 0.5 for acceptable construct indicators (Hair et al., 2010), ranging from 0.588 (COMS6: "*I felt that social status became irrelevant*") to 0.930 (COMS2: "*I felt a sense of belonging with the other participants*"). Internal consistency was further demonstrated by good Composite Reliability (CR = 0.84) and Average Variance Extracted (AVE = 0.63) across the COMS items. The mean reported total communitas (calculated as the sum across the 8 items) was 39.58 (SD = 11.23), corresponding to approximately 71% of the maximum score of 56. The average inter-item correlation was R = 0.602, individual bivariate correlations ranging from 0.415 to 0.870. In line with abovementioned factor loadings, the highest item-total correlation was observed for COMS2 (*r* = 0.901) and the lowest for COMS6 (*r* = 0.696), with an average item-total correlation of R = 0.807 (Supplementary Table S2).

#### Construct Validity

**FIGURE 3:**
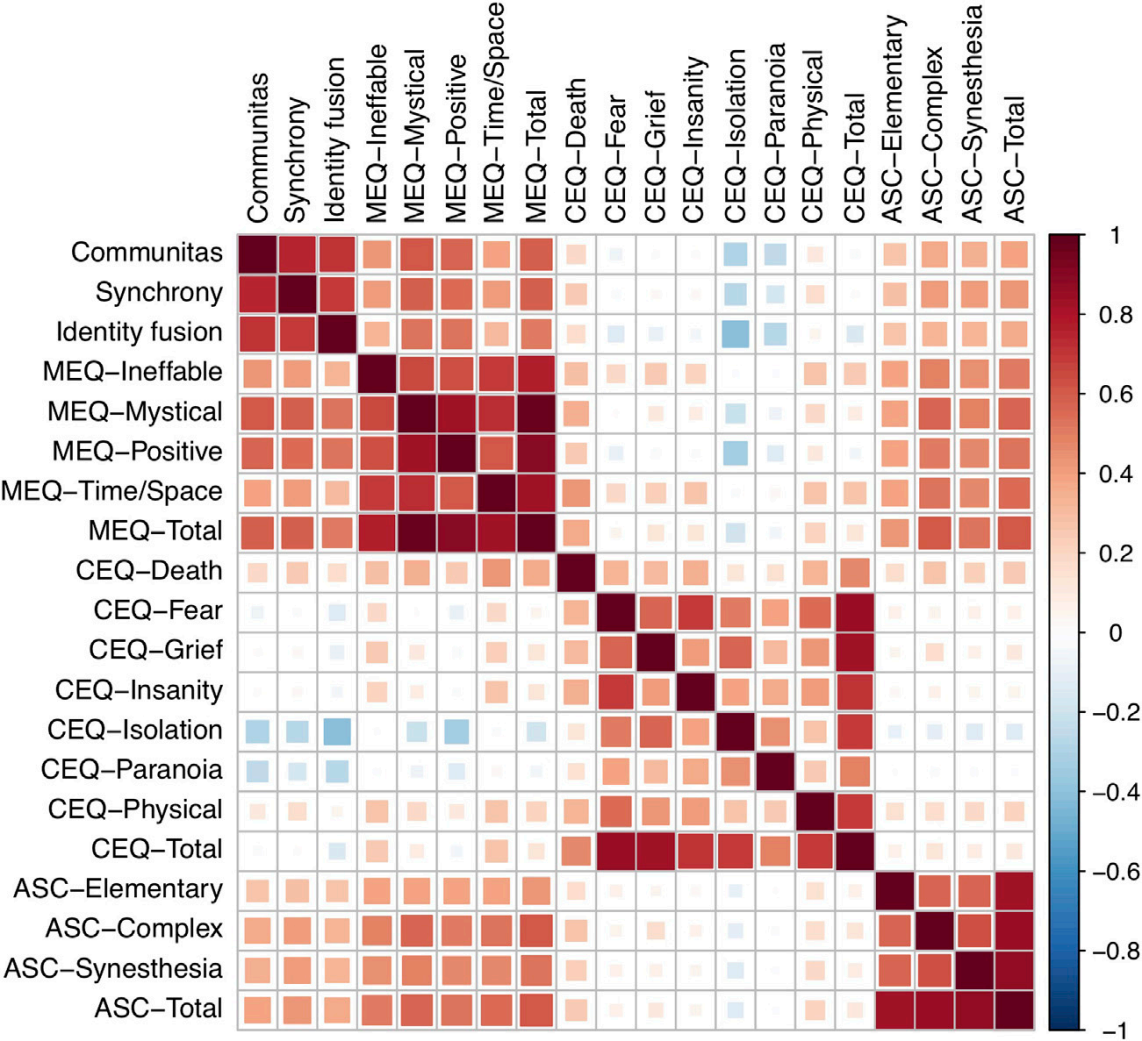
Correlation heatmap of subjective experience measures taken on the day after *N* = 720 psychedelic sessions. Color intensity and square sizes are both proportional to the (Pearson) correlation value. MEQ: Mystical Experience Questionnaire; CEQ: Challenging Experience Questionnaire; ASC: Visual experience subscales of the Altered States of Consciousness Questionnaire.

To assess construct validity, bivariate Pearson correlations were calculated between COMS scores and the total as well as subscale-scores of validated subjective experience measures taken after each ceremony (IF, PESC, MEQ, CEQ, ASC-VE; N in analysis = 720 observations). The results are displayed as a correlation heatmap in Figure 3.

The discriminant validity of the COMS was established against the other subjective experience measures and their subscales following the Fornell and Larcker (1981) criterion: namely, that the square root of the COMS′ average variance extracted ($\sqrt{AVE}$ = 0.79) was larger than the inter-construct correlation between the COMS and any of the other considered measures. Strong correlations were nonetheless found between the COMS and the two other measures of collective experience, i.e. identity fusion (IF, *r* = 0.71) and perceived emotional synchrony (PESC, *r* = 0.76). Moderate to strong positive correlations were also found between the COMS and the MEQ (*r* = 0.60), especially for the ‘Mystical’ (*r* = 0.61) and ‘Positive’ (*r* = 0.56) subscales. The COMS was furthermore positively correlated with visual aspects of the experience (ASC-Total), although to a lesser degree (*r* = 0.39). Lastly, weak negative correlations were discovered between the COMS and the ‘Isolation’ and ‘Paranoia’ subscales of the CEQ (*r* = −0.30 and r = −0.25, respectively).

**FIGURE 4:**
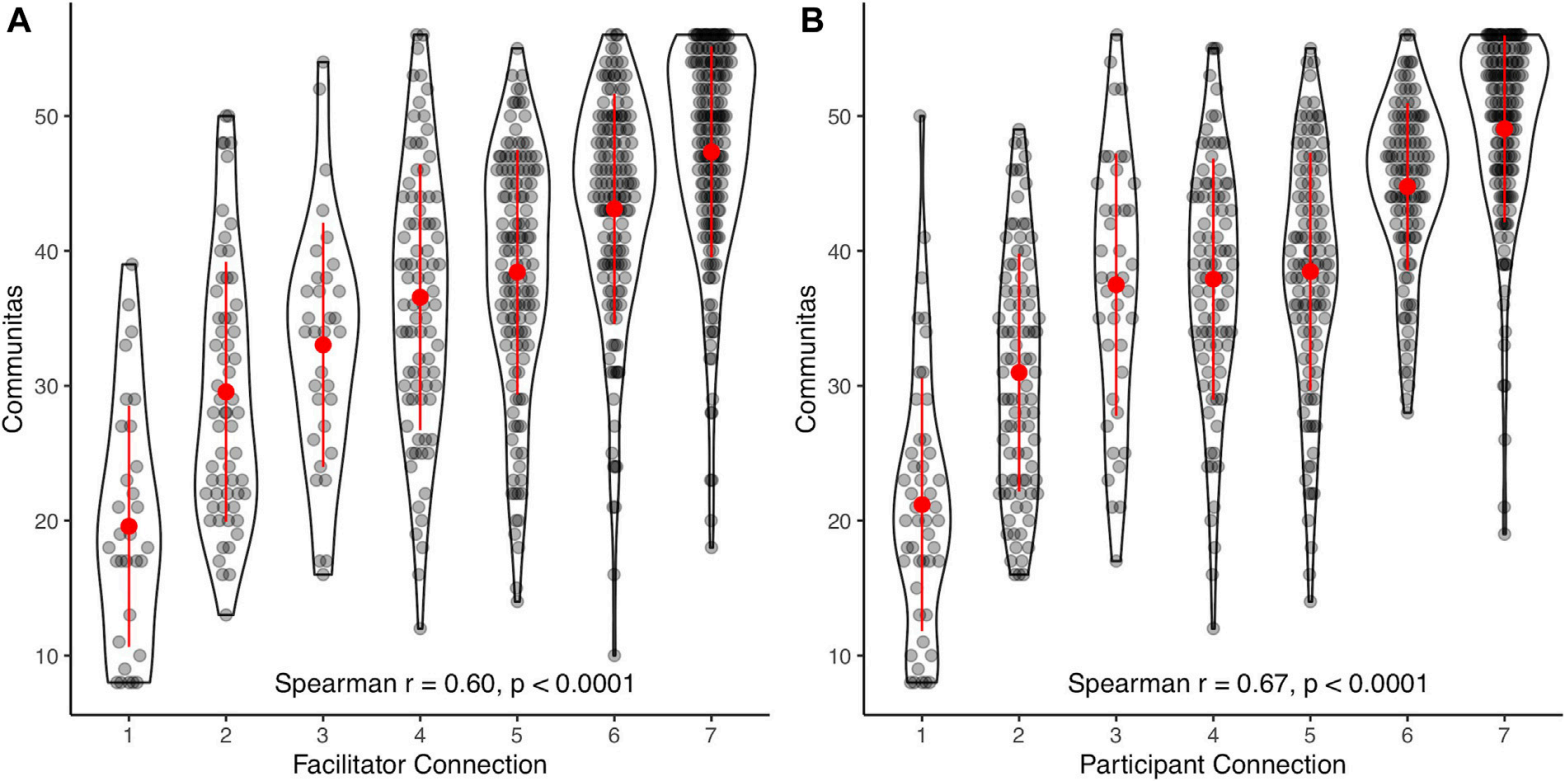
Distribution of communitas (COMS) scores across Likert-type responses on items measuring felt connection with a facilitator **(A)** or another participant **(B)** present during the psychedelic session.

The correlation between COMS scores and two additional Likert-scale items assessing perceived connection to 1) a facilitator and 2) another participant during the session were strong, with Spearman correlation of *r* = 0.60 (*p* < 0.0001), and *r* = 0.67 (*p* < 0.0001), respectively (Figure 4).

### Communitas Predicting Long-Term Outcomes

**FIGURE 5:**
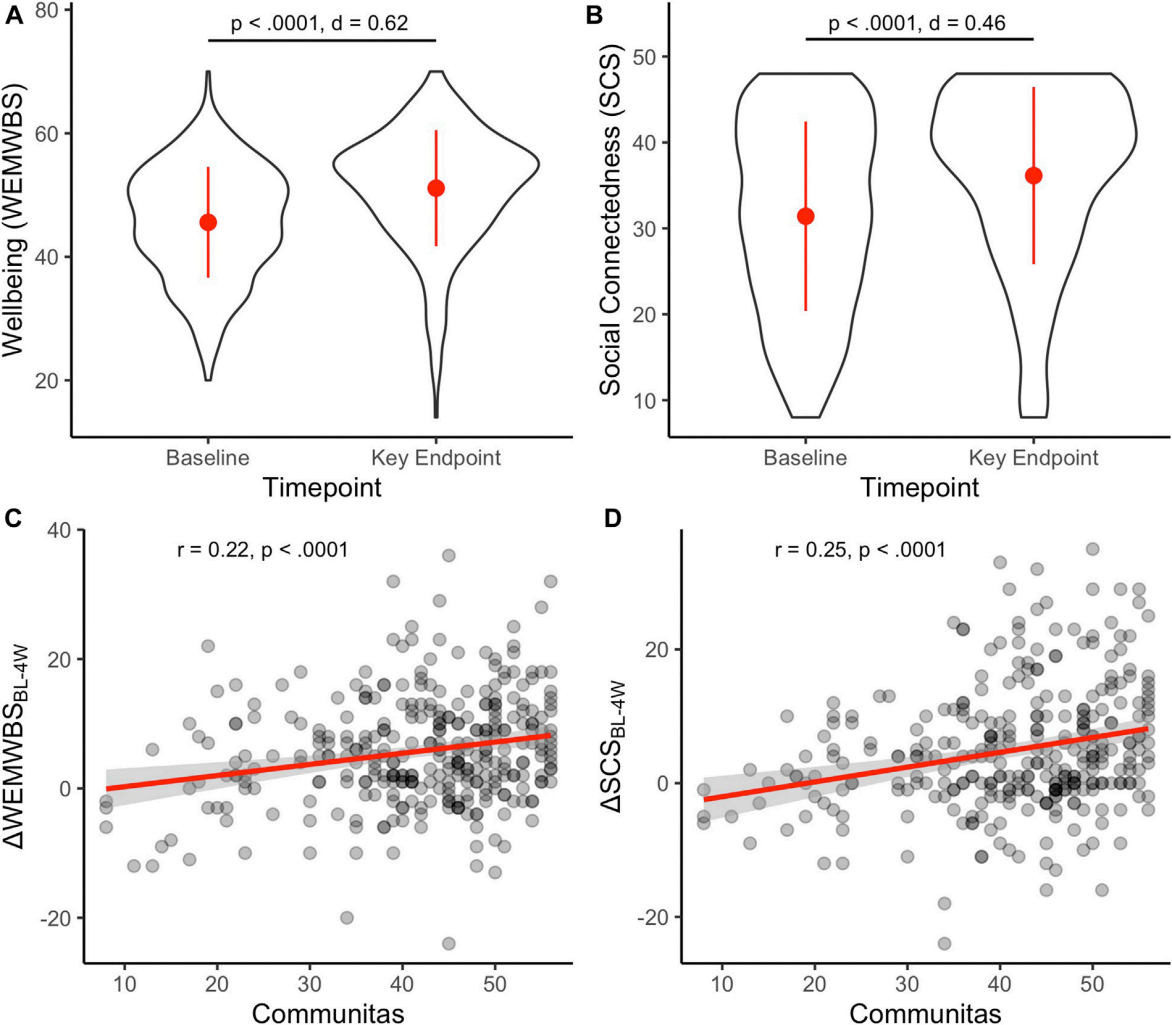
Post-psychedelic increases in psychological wellbeing (WEMWBS, **A)** and social connectedness (SCS, **B)** positively correlate with communitas **(C**, **D)** experienced during ceremony. Red dots and lines represent mean ± SD **(A, B)**, or trend lines **(C, D)**.

Two-sided paired t-tests showed that both social connectedness (SCS, M = 36.3, SD = 10.1) and wellbeing (WEMWBS, M = 51.2, SD = 9.2) were significantly higher at the 4 weeks endpoint than at baseline (Figures 5A,B, M = 31.5, SD = 11.1, t (373) = −9.9, *p* < 0.0001; M = 45.5, SD = 8.8, SD = 9.2, t (377) = −12.9, *p* < 0.0001; respectively), with substantial increases of medium and large effect sizes (Cohens d = 0.46 and 0.62, for SCS and WEMWBS). Wellbeing and social connectedness were significantly–albeit weakly—correlated with COMS scores experienced during the psychedelic session (Figures 5C,D; *r* = 0.22, and *r* = 0.25, respectively).

All secondary outcome variables changed significantly between baseline and key endpoint (see Table 4) with negligible (interpersonal tolerance, d = 0.19) to large (depressive symptoms, d = −0.60) effect sizes. Further establishing predictive validity of the COMS, communitas experienced during the psychedelic session was significantly, although weakly, correlated with changes on several of the secondary outcomes, including interpersonal tolerance (IPTS, *r* = 0.19), trait-anxiety (STAI, *r* = −0.16), and depressive severity (QIDS, *r* = −0.11).

**TABLE 4:** Changes on secondary outcomes, associations with communitas.

	Baseline (*N* = 819)		Key endpoint (*N* = 399)					Change scores vs. communitas	
	M	SD	M	SD	*t*	*p*	*d*	*r*	*p*
QIDS	6.98	4.77	4.20	3.33	−11.015	<0.0001	−0.60	−0.11	0.04
STAI	13.53	4.27	11.13	3.65	−11.745	<0.0001	−0.53	−0.16	<0.01
IPTS	5.57	0.74	5.69	0.68	5.043	<0.0001	0.19	0.19	<0.001

Means (M) and standard deviation (SD) are reported for each outcome at baseline (2 weeks before) and key endpoint (4 weeks after) a psychedelic retreat or ceremony. Significance and Cohen's d effect sizes are reported for two-sided pairwise t-tests, as well correlation coefficients with Communitas experienced during the psychedelic sessions. QIDS: Quick Inventory of Depressive Symptomatology; STAI: Trait version of the State-Trait Anxiety Inventory; IPTS: warm tolerance subscale of the Interpersonal Tolerance Scale.

#### Path Modeling

An initial longitudinal path model further exploring the causal role of communitas within psychedelic retreats was fitted including regressions paths between variables from a total of five time points (Figure 1). Listwise deletion showed better model fit than maximum-likelihood imputation of missing data, thus *N* = 213 subjects who completed all five time points were included in the analysis.

The initial model demonstrated less than acceptable fit on two of the four fit indices, namely the RMSEA and SRMR (Table 1). During the model pruning process, several variables were iteratively dropped due to lack of direct or mediated effects on communitas (COMS) or either of the outcomes SCS and WEMWBS: Identity Fusion (IF) measured before and after the ceremony, as well as after the retreat; Perceived emotional synchrony (PESC); Age. The final model exhibited good fit on all indices except the SRMR, where it nevertheless reached acceptable fit (Table 1).

**FIGURE 6:**
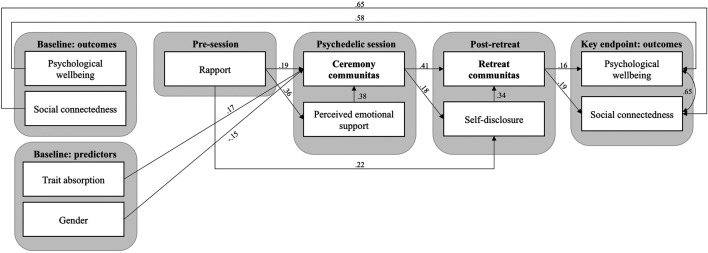
Final path model showing the structure of psychosocial mechanisms of psychedelic ceremonies, measured across 5 time points. Standardized coefficients are shown for significant (*p* < 0.05) regression paths.

In the final model (Figure 6), retreat communitas (i.e., communitas reported in reference to the retreat as a whole, rather than a specific ceremony), significantly predicted wellbeing and social connectedness 4 weeks later, with weak to moderate effect sizes (*β* = 0.16, *p* < 0.01 and *β* = 0.19, *p* < 0.001, respectively). Retreat communitas was, in turn, significantly predicted by communitas experienced acutely during the ceremony, both directly (*β* = 0.41, *p* < 0.0001), and indirectly mediated by self-disclosure that occurred over the course of the retreat (*β* = 0.18*0.34 = 0.06, *p* < 0.05). Ceremony communitas was influenced most strongly by rapport with facilitators and participants assessed hours pre-ceremony, both directly (*β* = 0.19, *p* < 0.01) and indirectly mediated by perceived emotional support during the ceremony (*β* = 0.36*0.38 = 0.14, *p* < 0.001). Trait absorption measured at baseline positively predicted ceremony communitas (*β* = 0.17, *p* < 0.01), while female gender was negatively associated with communitas (*β* = −0.17, *p* < 0.01). The standardized covariance between psychological wellbeing and social connectedness at the 4 weeks key endpoint was large (*r* = 0.65, *p* < 0.0001).

## Discussion

Through a series of controlled laboratory studies, the current resurgence of psychedelic research has produced a body of promising—albeit preliminary—evidence for the potential of psychedelic substances to alleviate mental health problems and enhance psychological wellbeing. Likely more so than with conventional psychopharmacological interventions, outcomes of psychedelic treatments are known to crucially rely on the acute subjective effects experienced by the user, which in turn, are affected by the psychological and environmental context of their use (Carhart-Harris et al., 2018c). Yet, the nature of modern testing settings has hindered so far the quantitative study of—what likely constitutes—one of the most longstanding and ubiquitous characteristics of naturalistic psychedelic use, i.e., the experience of psychedelic states as a collective, intersubjective activity. The present study constitutes a first step toward the quantitative assessment of psychosocial effects in guided psychedelic group experiences, enabled by the collection of the largest prospective sample of psychedelic use reported to date. By analyzing data from 720 individual psychedelic ceremony experiences reported by 886 participants assessed across 5 time points, we validated the proposed measure of communitas—an intense sense of togetherness and shared humanity—and clarified its relationships with existing measures of intra- and intersubjective phenomenology. Importantly, communitas was found to significantly predict enduring increases in psychological wellbeing and social connectedness following psychedelic use. Further examining the contextual determinants and downstream effects of communitas, a longitudinal path analysis revealed several psychosocial 'set and setting' predictors of communitas, as well as a mediating role of self-disclosure in extending communitas beyond the acute effects experienced during the psychedelic session.

In line with previous research using a similar measure of communitas in the context of nightclubbing (Taheri et al., 2016), good internal consistency, composite reliability, and convergent validity were established for communitas in the context of psychedelic group experiences. Confirming the conceptual link between the constructs, communitas was strongly associated with both perceived emotional synchrony and identity fusion, while still distinct enough to establish discriminant validity and thus demonstrate the new measure's additional value according to the Fornell and Larcker (1981) criterion. Perceived emotional synchrony (also known as 'collective effervescence') experienced during collective events such as folkloric festivals, protest demonstrations, mindful dancing, and sport activities, has been shown to enhance collective identity, social cohesion, personal and collective self-esteem and efficacy, positive affect, positive social beliefs, compassion, and individual wellbeing (Páez et al., 2015; Pizarro et al., 2020; Wlodarczyk et al., 2020). In a similar vein, the present study provides novel evidence of enduring prosocial and psychological benefits derived from the experience of communitas during a collective psychedelic experience. In particular, post-psychedelic improvements in wellbeing, social connectedness, depressive symptoms, trait anxiety, and interpersonal tolerance, were found to be positively associated with the extent of communitas experienced during psychedelic ceremonies, demonstrating the predictive (criterion) validity of the here proposed instrument. This finding adds to quantitative studies on collective activities without a pharmacological component that have demonstrated a role of communitas in the formation of collective identity (Lee et al., 2015), experiential involvement (Taheri et al., 2016), volunteer engagement and commitment (Curran and Taheri, 2019), and repeat attendance of events (Jahn et al., 2018; Lee et al., 2015; McGinnis et al., 2008), but not yet shown any enduring improvements of psychological and social health.

The observed increases in psychological wellbeing are in accordance with previous research, which indicates that guided and ceremonial use of psychedelics can lead to long-lasting psychologically beneficial changes. Experiences of psychedelic ceremonies have been consistently reported as positive, valuable, and in many cases associated with health improvements by both novice and regular participants (Barbosa et al., 2009; Loizaga-Velder and Verres, 2014). Furthermore, in line with the current results, previous observational studies on psychedelic ceremony participants have found increased wellbeing (Bouso et al., 2012; Uthaug et al., 2018b; Spriggs et al., 2020), creative divergent thinking (Kuypers et al., 2016; Mason et al., 2019), cognitive flexibility and mindfulness-related capacities (Soler et al., 2016; Soler et al., 2018; Murphy-Beiner and Soar, 2020; Zeifman et al., 2020), as well as reduced abuse of alcohol and other addictive drugs (Doering-Silveira et al., 2005). It is worth noting that absolute change (ΔWEMWBS = 5.7 in the current sample vs. 2.8 reported in Haijen et al., 2018) and effect size (d = 0.62 vs. d = 0.4, reported in Roseman et al., 2019) for post-psychedelic wellbeing increase were approximately 2 and 1.5 times higher in the current study than in a structurally similar sample that considered naturalistic psychedelic use unrestricted to ceremony settings, suggesting that psychedelics used in guided, collective environments may on certain occasions provide an added advantage for the prevention and treatment of mental health problems.

Large observed decreases in depression severity, despite a potential floor-effect,^2^ constitute a meaningful parallel to recent controlled trials using ayahuasca or psilocybin in clinical settings, including the treatment of drug dependence (Bogenschutz et al., 2015; Johnson et al., 2014; Thomas et al., 2013), depression (Carhart-Harris et al., 2016; Palhano-Fontes et al., 2018), and end-of-life anxiety (Grob et al., 2011; Griffiths et al., 2016; Ross et al., 2016). Pooling data from multiple observational samples, including the one reported here, Spriggs et al. (2020) could establish first preliminary evidence of post-psychedelic improvements in a clinically relevant population yet unexplored in context of psychedelic treatments, i.e. those reporting an eating disorder. Acknowledging differences in population characteristics and needs between ceremony participants and patients enrolled in clinical trials, these findings thus strongly indicate collective phenomena as bearing underexploited potential for psychedelic therapy, warranting further research into the potential clinical utility of psychedelic retreats and psychedelic-assisted group therapy approaches.

The strongest association between any long-term outcome and communitas was observed for social connectedness, pointing to the ability of positive relational experiences during psychedelic ceremonies to induce a sense of belongingness beyond the context of the immediate social environment. Social connectedness is a well-established determinant of both mental and physical health (Alexander, 2010; Hari, 2019), with large-scale studies and meta-analyses reliably showing social isolation and loneliness to be on par with obesity and heavy smoking in their effects on mortality (Holt-Lunstad et al., 2010; Pantell et al., 2013; Rico-Uribe et al., 2018). The capacity of psychedelic use to enduringly enhance feelings of social connectedness has (to our knowledge) only been shown in one other prospective study, which was similar in design but not restricted to participants in ceremony settings (Carhart-Harris et al., 2018a). Further underlining the capacity of guided collective environments of psychedelic use to instil lasting positive psychosocial effects, the current sample of showed increases in social connectedness that were on average 2.7 times higher than in a methodologically comparable naturalistic study (ΔSCS = 4.8 vs. 1.8, as reported in Carhart-Harris et al., 2018a) unrestricted to ceremonial environments.

Social connectedness and wellbeing measured at the key endpoint covaried highly, in line with recent findings from psychedelic users at mass gatherings where social connectedness partly mediated the effects of psychedelic use on positive mood (Forstmann et al., 2020), assessed cross-sectionally. Mechanistically, it is possible that psychedelics enhance social connectedness by improving socio-cognitive functioning, considering that experimental studies have found increased emotional empathy following psychedelic use (Dolder et al., 2016; Pokorny et al., 2017; Hutten et al., 2019), as well as attenuated feelings of social exclusion and social rejection processing in the brain (Preller et al., 2016). The current finding of enhanced interpersonal tolerance 4 weeks following a psychedelic ceremony or retreat adds to this body on psychedelic-induced alterations of social processing, providing what is—to our knowledge—the first evidence of a socio-pharmacological intervention capable of fostering more permissive and accepting attitudes toward others whose opinions, beliefs, practices, or values, differ from one's own. It is conceivable that the ability of psychedelics to induce more liberal political attitudes (Nour et al., 2017; Lyons and Carhart-Harris, 2018) may be related to this increase in interpersonal tolerance, a mechanism that we plan to investigate further in the context of inter-group conflict (Roseman et al., 2021).

In line with the predictive model of naturalistic psychedelic use provided by Haijen et al. (2018), the acute experience of communitas in the current sample was predicted by several psychological and contextual determinants, most significantly by perceived emotional support during ceremony, which in turn mediated some of the effects of rapport with group and facilitators rated hours before the session. The establishment of a supportive social environment and trust among participants, as well as between participants and facilitators, should therefore be considered a priority for approaches to collective psychedelic use that aim to enhance psychological wellbeing and social connection. Importantly, while communitas experienced during ceremony was in itself correlated with positive outcomes, the path model revealed that all enduring positive effects of ceremonial communitas were mediated by the experience of communitas reported in relation to the retreat as a whole (i.e., beyond a specific ceremony). Hence, the enduring benefits of communitas were fully explained through the expansion of this positive social experience beyond the acute psychedelic state. Importantly, this extension of communitas beyond the ceremony was partly mediated by self-disclosure, i.e., how deeply and honestly people shared personally salient material with the group. The establishment of social devices that facilitate emotional disclosure, such as sharing rounds, thus measurably contributed to positive outcomes following collective psychedelic experiences, at least in the current sample consisting mostly of psychedelic retreat participants.

Together, these findings expand current models that place individual experience as a central psychological mechanism for the attainment of mental health outcomes, by supporting the notion that ‘experiencing with others’ may further enhance their therapeutic action. By making explicit the association between molecular and psychosocial mechanisms of therapeutic action, psychedelics appear to be particularly meaningful tools to render visible the embodied, embedded, and (possibly) extended character of cognition, affect, and mental health (Ward and Stapleton, 2012).

### Study Limitations

The web-based observational design of the current study entailed several intrinsic limitations. Firstly, the lack of experimental control meant prevent confirming whether participants completed questionnaires at the correct time points in reference to their experience, and verifying under what conditions these measures were completed. Additionally, the substantial number of participants dropping out from the study before completing the key endpoint may have introduced a systematic attrition bias, although soon to be published analyses on the sample described by Haijen et al. (2018) suggest that predictors of attrition in observational psychedelic research may be no more problematic than in other fields of research (Hübner et al., in prep). Moreover, the setting considered in this study attracted a predominantly WEIRD (white, educated, industrialized, rich, democratic) (Henrich et al., 2010), self-selected sample, reflecting a pervasive problem in psychedelic research (George et al., 2020), which in the current case may have been accentuated by material barriers to the participation in—often costly—retreats. Consequently, the cross-cultural validity of the here proposed communitas scale remains unclear, if not doubtful, given the limited cultural translatability of concepts such as 'ego', invoked in some of the items. The sample characteristics also call into question to what extent biases toward the positive effects of psychedelics may have led to demand characteristics influencing study outcomes–which should be taken into account by addressing participant expectations and biases in future studies. Furthermore, heterogeneous approaches to the preparation, administration, and communication of drug doses in ceremony settings made it impossible to control for the quantity or potency of substance consumed by participants, which we hope to improve in future studies. Lastly, retreat settings in remote locations may, in some cases, have been visited by participants as part of more extensive journeys, meaning that the assessed psychological variables may have been confounded by additional travel experiences, which are known to positively affect mental and physical health (Chen and Petrick, 2013).

### Directions for Future Research

With an increasing number of completed clinical studies and growing datasets of naturalistic psychedelic use, a salient question for future research lies in the comparison of the two. Acknowledging the obvious safety benefits of highly controlled clinical and laboratory environments, the current findings suggest that certain elements in naturalistic settings, in particular their collective social dimension, may hold a unique potential for enhancing positive outcomes of psychedelic use. More detailed assessment of clinically relevant variables will allow future studies to expand on the current results by exploring the therapeutic potential of collective experiences in populations suffering from specific mental illnesses. Specifically in the case of posttraumatic stress for example, authors have discussed the value of therapeutic rituals (Johnson et al., 1995), self-disclosure (Bowen et al., 2010), and strong communities (Bracken, 2002). While observational studies on psychedelic effects will not replace randomized controlled trials, their findings may inform new treatment avenues by uncovering psychological mechanisms that could not be explored in smaller samples and including new patient populations that have not yet been treated with psychedelics in clinical settings (see e.g., Spriggs et al., 2020). In order to enhance the validity of this line of research, and further explore the sociophysiological underpinnings of psychedelic experiences, assessment of biometric markers such as heart rate dynamics should be considered, which are known to synchronize during intense collective events (Xygalatas et al., 2011). Additionally, the cross-cultural validity of the here proposed measure of communitas should be tested within non-WEIRD populations, especially considering that the concept of communitas has been developed through the study of rituals in African tribal (i.e., non-WEIRD) communities (Turner, 1969). It is likely that the experience of communitas would have different roles and effects in members of non-WEIRD cultures, where frameworks of psychedelic use can be strongly divergent from common Western narratives of individual psycho-spiritual healing or growth (for a discussion in the context of Peruvian ayahuasca use, see Losonczy and Cappo, 2014); including for example the use of ayahuasca to perform aggressive actions toward hostile shamans or individuals through means of sorcery or witchcraft (Wilbert and Vidal, 2004; Fotiou, 2016).

One could conjecture that psychedelic serotonin 2A agonists may uniquely facilitate the emergence of communitas, for example through a temporary, substance-specific weakening of high-level (social) cognitive structures in the brain (Carhart-Harris and Friston, 2019), thereby promoting a mode of intersubjective cognition unconstrained by social roles and hierarchies and thus conducive to liminality. Indeed, psychedelics have been shown to decrease connectivity between areas of the default mode network (Carhart-Harris et al., 2014) including the hippocampus and medial prefrontal cortex, the selective coupling of which has recently been implicated in the updating and representation of self-relevant social hierarchies (Kumaran et al., 2016). This hypothesis could be tested in future research through controlled assessment of similar rituals that do not involve psychedelic use. Systematic assessment of environmental factors present in such settings would allow for a more detailed understanding of the constituents within psychedelic and non-psychedelic ritual environments that elicit communitas and related psychosocial experiences, which would be of great scientific and medical relevance. Lastly, psychedelic-induced increases in suggestibility (Carhart-Harris et al., 2015) and perceived meaning (Hartogsohn, 2018), raise the ethically complex question to what extent culturally rich ceremonial environments of psychedelic use may lead to a transmission of beliefs and behaviors (Dupuis, 2016; Dupuis, 2020), a question that we are currently studying through the assessment of post-psychedelic changes in belief structures (Timmermann et al., 2018). As an extreme negative case, it is conceivable how enhanced suggestibility, paired with a felt dissolution of boundaries between the self and a social environment functioning under radically different cultural or cosmological priors, apart from immediate risks such as sexual abuse (the prevalence of which has been pointed out recently by Peluso et al., 2020), could lead to psychological destabilization, or unconscious and thus not fully consensual conversion experiences (Dupuis, 2018). The consequential need for grounded practices for the mediation and validation of psychedelic insights has recently been discussed by Timmermann et al. (2020).

### Implications: Toward a Biopsychosocio-Cultural Approach to Psychedelics

With psychedelic-assisted psychotherapies, a new modality is presently finding its way into the arsenal of psychotherapeutic and pharmacological interventions that psychiatry wields to maintain and restore psychological health. While connectedness has already emerged into the focus of psychedelic therapy (Watts et al., 2017; Carhart-Harris et al., 2018a), we hope that the present findings may serve as an encouragement for the continued study of psychedelics within a biopsychosocio (-cultural) framework (George and Engel, 1980; Hilty, 2015), not merely as psychopharmacological agents that can enhance individual wellbeing and personal growth, but may do so by fostering a sense of community, interpersonal trust and tolerance, expanding their potential for positive change beyond the level of the individual.

Apart from improving accessibility through reduction of treatment costs, group settings that focus on relationships and community, rather than solely individual processes, might constitute one approach to tackle the problem of transcultural applicability of psychedelic treatments. As Kirmayer and Young (1998) note: "For people from many cultures, the harmony of the family and the group is more important than individual autonomy […] Fostering individualism through psychotherapy may put people from such cultures more at odds with their families and local worlds and so undermine both social support and their own sense of self-worth. As a result, solutions that make sense from the perspective of Euro-American psychiatry and health psychology may involve tradeoffs for some ethnocultural groups that negate even the presumptively universal mechanisms of catharsis and healing". The same will likely also apply to the emerging field of psychedelic-assisted therapies, where emotional catharsis is seen as an important treatment mechanism while ethnic disparities rooted in exclusionary sociocultural narratives of Western individualism prevail (George et al., 2020; Williams and Labate, 2020). Amidst the imminent pharmaceuticalization of psychedelics (Noorani, 2020), we hope that the present study may thus draw attention to the specific capacities in which collective psychedelic use, historically prominent in underground, indigenous and sacramental contexts, may provide benefits for individuals and collectives, despite being mostly neglected by current research–emphasizing the inherent value in a pluralistic ecosystem of approaches to psychedelic use, as opposed to conventional one-size-fits-all medicalized treatment. In order for medical psychedelic use to accommodate the variability in individual needs and responsivity within a precise-personalized approach (Kelly et al., 2020), we propose that innovative trial designs and methods will be necessary, including pragmatic trials collecting large real-world data and aggregated single-case studies based on idiographic and high-frequency assessment of outcomes, e.g. through mobile applications (Carhart-Harris et al., forthcoming).

Even though the present results suggest positive prosocial effects of collective psychedelic use, qualitative studies have found a tendency, in neoshamanic settings of ayahuasca use (Gearin, 2015b; Gearin, 2016a; Rodd, 2018), for individualistic and narcissistic cultural priors to be reproduced in ideas of self-actualization and -development, which are similarly defining for New Age culture (Graf, 1986; Farias and Lalljee, 2008; Lahood, 2010; Masters, 2010) as they are for neoliberalist-entrepreneurial constructions of selfhood (Adams et al., 2019). In order for psychedelics to produce the healthy paradigm shift in psychiatry that some have predicted (Nichols et al., 2017; Schenberg, 2018), it may thus be necessary to question some of the fundamental cultural assumptions from, and into which psychedelic therapies are emerging–so that psychedelic treatments may not merely remain a "*chemical holiday* […] into a strictly private special sphere" (Martin Buber, 1965 [*p*. 100], on Aldous Huxley's mescaline experiments), but instead, foster meaningful connections within relationships and communities, the importance of which have perhaps seldom been clearer than during the social disruption currently caused by the COVID-19 pandemic.

*Beneath the surface we are connected* (Tempest, 2020, p. 48)

## Conclusion

Historically and contemporarily, the use of psychedelic substances in real-world settings rarely happens in isolation, but rather, as a shared experience. By leveraging a large prospective sample of psychedelic ceremony participants, the present study revealed the relevance of the psychosocial context and experience during, before, and after psychedelic sessions for predicting their lasting psychological effects. Specifically, the study introduced and psychometrically validated a novel measure of spontaneous communitas in the context of psychedelic experiences, denoting a state of “togetherness” and shared humanity between participants that was shown to induce enduring benefits to wellbeing and social connectedness. These findings emphasize the value of psychosocial approaches in psychedelic research, which can provide a complementary perspective to the growing body of literature concerned with the psychological mechanisms underlying psychedelic use.

## Data Availability

The raw data supporting the conclusion of this article will be made available by the authors, without undue reservation.

## Footnotes

1 In case of subjects who participated in multiple psychedelic sessions over the course of a longer retreat, time points 1-3 were repeated for each completed session.

2 The average baseline depression severity being close to the bottom end of a 'mild depression', with 6.98 vs. threshold of 6.0, suggested by Rush et al. (2003).

## Appendix

### The adapted psychedelic Communitas Scale (COMS)

Please indicate how strongly you agree or disagree with the following statements about your past experience, taking into account that 1 = Strongly disagree and 7 = Strongly agree.

**Table:**

1 = Strongly disagree, 2 = Disagree, 3 = Somewhat disagree, 4 = Neither agree nor disagree, 5 = Somewhat agree, 6 = Agree, 7 = Strongly agree							
During the ceremony, I felt a bond with my fellow participants that I could not experience outside the ceremony	1	2	3	4	5	6	7
During the ceremony, I felt a sense of belonging with the other participants	1	2	3	4	5	6	7
During the ceremony, I felt a sense of harmony with the others	1	2	3	4	5	6	7
During the ceremony, I felt a sense of sharing with the other participants	1	2	3	4	5	6	7
The ceremony really allowed me to get to know the other participants	1	2	3	4	5	6	7
During the ceremony, I felt that social status became irrelevant	1	2	3	4	5	6	7
During the ceremony, I experienced all participants as equal based on basic shared humanity	1	2	3	4	5	6	7
During the ceremony, ego-related tensions between participants seemed to resolve	1	2	3	4	5	6	7
During the ceremony, I felt a strong connection to another participant	1	2	3	4	5	6	7
During the ceremony, I felt a strong connection the facilitator or shaman	1	2	3	4	5	6	7

For use post-retreat, every instance of 'ceremony' was replaced with ‘retreat' (e.g., “The retreat really allowed me to get to know the other participants.”
