# Supplementary material for: Psychedelic Communitas: Intersubjective Experience During Psychedelic Group Sessions Predicts Enduring Changes in Psychological Wellbeing and Social Connectedness
Source: Front Pharmacol. 2021 Mar 25;12:623985. doi: 10.3389/fphar.2021.623985 (PMC8114773; doi:10.3389/fphar.2021.623985)
Supplement: Supplementary file 1 [file datasheet1.docx]

Supplementary Material

# Supplementary Figures and Tables

## Supplementary Figures


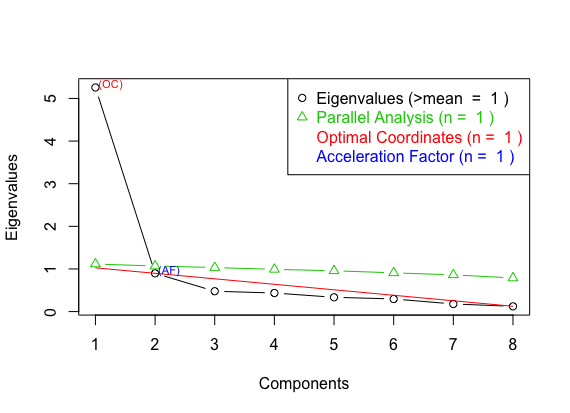


**Supplementary Figure 1.** Scree plot displaying eigenvalues and results of non-graphical tests to determine optimal number of factors to retain in the adapted Communitas Scale (COMS).

| 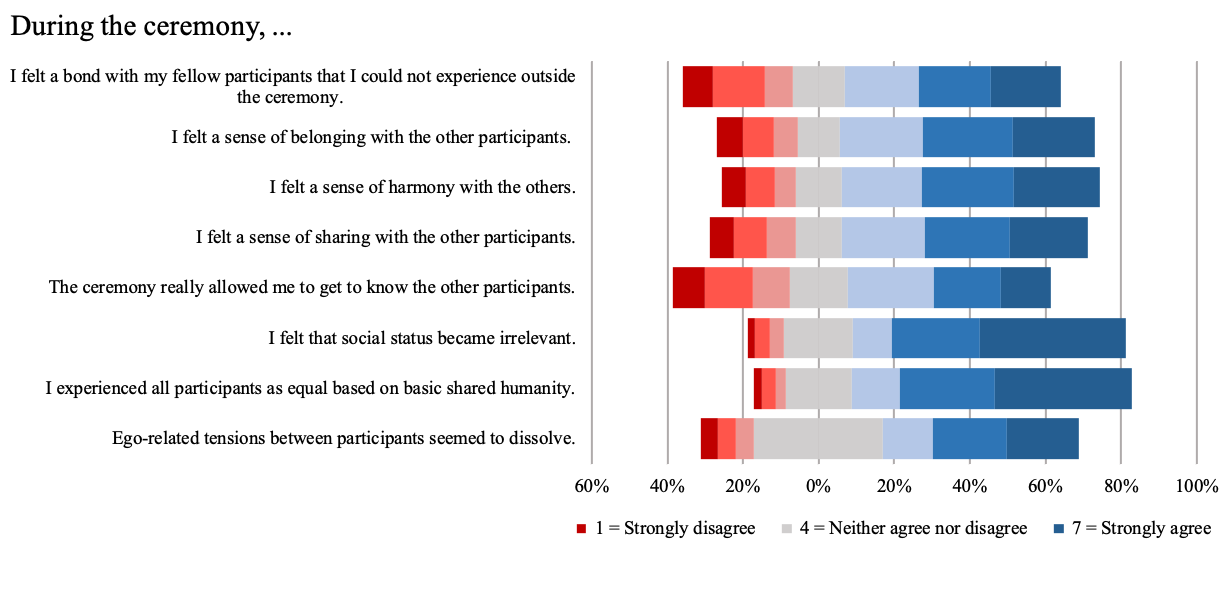 |
| --- |
| **Supplementary Figure 2.** Item distributions of the adapted Communitas Scale (COMS). |

## Supplementary Tables

| Supplementary Table 1  *Demographic information of participants who have completed time point 3 (1 day post-ceremony)* | | |
| --- | --- | --- |
| **Total N** |  | **495** (of 533*) |
| Age |  | 44.3 ± 12.2 |
| Gender | Female | 220 (44.4%) |
|  | Male | 272 (55.0%) |
|  | Other | 3 (0.6%) |
| Nationality | United States | 197 (39.8%) |
|  | United Kingdom | 105 (21.2%) |
|  | Australia | 17 (3.4%) |
|  | Germany | 17 (3.4%) |
|  | Canada | 18 (3.6%) |
|  | Other countries (52 in total) | 141 (25.5%) |
| Education | None | 4 (0.8%) |
|  | High School or equivalent (GED) | 27 (5.5%) |
|  | Associate / Technical Degree | 36 (7.3%) |
|  | College Diploma | 151 (30.5%) |
|  | Master’s Degree | 170 (34.3%) |
|  | Doctorate or Professional Degree | 107 (21.6%) |
| Employment | Student | 28 (5.6%) |
|  | Working full-time | 316 (63.8%) |
|  | Working part-time | 77 (15.6%) |
|  | Retired | 42 (8.5%) |
|  | Unemployed | 32 (6.5%) |
| Median household income |  | 8000 $ |
| Ethnicity | White | 456 (92.7%) |
|  | Black or African American | 7 (1.2%) |
|  | Asian | 26 (5.3%) |
|  | American Indian or Alaska native | - |
|  | Unknown / Prefer not to say | 2 (0.4%) / 16 (3.2%) |
| Marital status | Cohabiting with partner | 55 (11.1%) |
|  | Married | 209 (42.2%) |
|  | Divorced | 48 (9.7%) |
|  | Separated | 20 (4.0%) |
|  | Never married | 155 (31.3%) |
|  | Widowed | 8 (1.6%) |
| Previous psychedelic use | Never | 204 (41.2%) |
|  | Once | 53 (10.7%) |
|  | 2-5 times | 104 (21.0%) |
|  | 6-10 times | 43 (8.7%) |
|  | 11-20 times | 44 (8,9%) |
|  | 21-50 times | 29 (5.9%) |
|  | > 50 times | 18 (3.6%) |
| *Note.* Median value is given for *Income.* For *Age,* mean ± standard deviation is shown. Absolute frequencies including corresponding percentages (in brackets) are presented for other items. Multiple responses were possible for *Ethnicity*  **38 participants completed time point 3, but not baseline, and thus have no demographic information available.* | | |

| Supplementary Table 2  *Inter-item and item-total correlations of the Communitas Scale* | | | | | | | | | |
| --- | --- | --- | --- | --- | --- | --- | --- | --- | --- |
| Item | COMS1 | COMS2 | COMS3 | COMS4 | COMS5 | COMS6 | COMS7 | COMS8 | COMS Total |
| COMS1 | 1.000 |  |  |  |  |  |  |  | 0.833 |
| COMS2 | 0.790 | 1.000 |  |  |  |  |  |  | 0.901 |
| COMS3 | 0.743 | 0.870 | 1.000 |  |  |  |  |  | 0.897 |
| COMS4 | 0.714 | 0.816 | 0.830 | 1.000 |  |  |  |  | 0.875 |
| COMS5 | 0.603 | 0.638 | 0.629 | 0.665 | 1.000 |  |  |  | 0.763 |
| COMS6 | 0.450 | 0.518 | 0.531 | 0.487 | 0.415 | 1.000 |  |  | 0.696 |
| COMS7 | 0.498 | 0.587 | 0.592 | 0.550 | 0.440 | 0.639 | 1.000 |  | 0.750 |
| COMS8 | 0.517 | 0.559 | 0.567 | 0.550 | 0.489 | 0.546 | 0.630 | 1.000 | 0.742 |
| Average value of the inter-item correlations: R=0.602; Average value of the item-total correlations: R=0.807 | | | | | | | | | |
